# Comparative genomics of 43 strains of *Xanthomonas citri* pv. *citri* reveals the evolutionary events giving rise to pathotypes with different host ranges

**DOI:** 10.1186/s12864-015-2310-x

**Published:** 2015-12-23

**Authors:** Jonathan L. Gordon, Pierre Lefeuvre, Aline Escalon, Valérie Barbe, Stéphane Cruveiller, Lionel Gagnevin, Olivier Pruvost

**Affiliations:** Université de la Réunion, UMR PVBMT, 97410 Saint-Pierre, La Réunion France; CIRAD, UMR PVBMT, 97410 Saint-Pierre, La Réunion France; CEA/DSV/IG/Genoscope, 2 rue Gaston Crémieux, BP5706, 91057 Evry, France; Current Address: CIRAD, UMR CMAEE, F-97170 Petit-Bourg, Guadeloupe France; Current Address: UMR IPME, IRD-CIRAD-Université Montpellier, 34394 Montpellier, France

**Keywords:** Xanthomonas citri, Plant pathogen, Genome evolution, Pathotype evolution, Host range determination, Recombination, Gene islands, Ancestral character estimation, Pathogenicity, Gene presence/absence

## Abstract

**Background:**

The identification of factors involved in the host range definition and evolution is a pivotal challenge in the goal to predict and prevent the emergence of plant bacterial disease. To trace the evolution and find molecular differences between three pathotypes of *Xanthomonas citri* pv. *citri* that may explain their distinctive host ranges, 42 strains of *X. citri* pv. *citri* and one outgroup strain, *Xanthomonas citri* pv. *bilvae* were sequenced and compared.

**Results:**

The strains from each pathotype form monophyletic clades, with a short branch shared by the A^w^ and A pathotypes. Pathotype-specific recombination was detected in seven regions of the alignment. Using Ancestral Character Estimation, 426 SNPs were mapped to the four branches at the base of the A, A*, A^w^ and A/A^w^ clades. Several genes containing pathotype-specific nonsynonymous mutations have functions related to pathogenicity. The A pathotype is enriched for SNP-containing genes involved in defense mechanisms, while A* is significantly depleted for genes that are involved in transcription. The pathotypes differ by four gene islands that largely coincide with regions of recombination and include genes with a role in virulence. Both A* and A^w^ are missing genes involved in defense mechanisms. In contrast to a recent study, we find that there are an extremely small number of pathotype-specific gene presences and absences.

**Conclusions:**

The three pathotypes of *X. citri* pv. *citri* that differ in their host ranges largely show genomic differences related to recombination, horizontal gene transfer and single nucleotide polymorphism. We detail the phylogenetic relationship of the pathotypes and provide a set of candidate genes involved in pathotype-specific evolutionary events that could explain to the differences in host range and pathogenicity between them.

**Electronic supplementary material:**

The online version of this article (doi:10.1186/s12864-015-2310-x) contains supplementary material, which is available to authorized users.

## Background

Bacteria from the genus Xanthomonas are major phytopathogens of a wide variety of plants and represent species of great agricultural and economic importance [1]. In general, *Xanthomonas* species have restricted host ranges, each only specializing in the infection of a small number of plant species [2]. *Xanthomonas citri* pv. *citri* (previously *X. axonopodis* pv. *citri*) is a pathogenic bacterium that infects citrus plants and is the global cause of Asiatic citrus canker [3], resulting in significant crop losses around the world and giving *X. citri* pv. *citri* the status of a quarantine organism in some countries that do not face it [3]. *X. citri* pv*. citri* invades citrus plants through the stomata or wounds and attacks the plant cells with a range of different virulence factor proteins transported out of the bacterial cell by the Type II-Type VI secretion systems [4]. Both the pathogen and its host species originate from Asia [2, 5].

Strains of *Xanthomonas citri* pv. *citri* were subdivided into different pathotypes based on their host specificity and the defense response to infection by different citrus host species. To date there have been three main groups identified, designated A, A* and A^w^ pathotypes [6, 7]. Pathotype A has the broadest host range, infecting most *Citrus* species and related genera and is the most agriculturally important of the pathotypes. Conversely, A* and A^w^ have very limited host ranges and have only been isolated from Key lime (Citrus aurantifolia) and alemow (C. macrophylla). The A* strains were isolated from Key lime in several countries in Asia, and were recognized as a distinct pathotype from the A strains due to their inability to develop canker lesions on grapefruit [7, 8]. The A^w^ pathotype differ from the A* strains in their ability to elicit a hypersensitive response (HR) on grapefruit and sweet orange [6]. The *avrGf1* (*syn. xopAG*) gene that hasn’t yet been found in the other pathotypes than A^w^ is at least partly responsible for HR in grapefruit and sweet orange [9–11]. Deletion reduces the HR symptoms in grapefruit and sweet orange, but doesn’t increase the host range of the strain indicating a more complex determination of host range. Transconjugation of *avrGf1* or its homolog *avrGf2* into A strains elicits a HR in grapefruit, indicating that it can act as a host range restriction factor, even if it isn’t the primary cause of A^w^ host range restriction [12].

Identifying the underlying causes for the different host ranges of the three closely related *X. citri* pv. *citri* pathotypes may provide new targets to aid in the prevention of bacterial diseases and possibly inform new strategies to treat and manage citrus canker outbreaks. Most notably, it would also allow the improvement of our understanding of how bacterial pathogens evolve in terms of host range variations when coevolving with their host(s) in agrosystems, and more generally on plant bacterial disease emergence [13]. Here we use NGS data from 42 strains of X. citri pv. citri representing the three pathotypes and selected on the basis of our current knowledge of the bacterium’s genetic diversity as well as one outgroup strain of *X. citri* pv. bilvae (also pathogenic to rutaceous species but with a distinct symptomatology [14]), to construct a phylogeny using aligned non-recombinant genomic regions from all strains. Based on this phylogeny, we identify pathotype-specific genomic changes from the level of single base changes to the level of multi-gene islands and regions of recombination.

## Results

### Sequencing

**Fig. 1:**
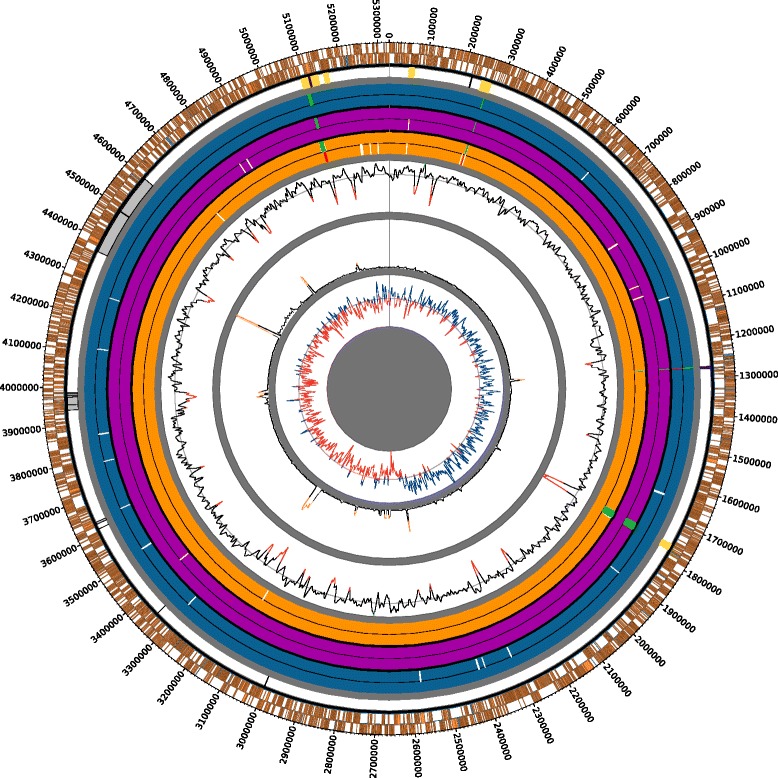
Circular map representing the genome alignment of 43 *X. citri* and 1 *X. bilvae* strain. The outermost tracks depict the protein-coding (*orange*) and RNA (*blue*) genes found on the forward (*outer*) and reverse (*inner*) strands of X. citri strain 306 that have been mapped onto the alignment. The next innermost track represents the regions of detected recombination in A^w^ (*yellow*), A* (*dark blue*) and non pathotype-specific events (*grey*). Further towards the centre, the gene islands and pseudogenes for A* (*dark blue*), A (*dark purple*), and A^w^ (*dark brown*) pathotypes are show respectively. Each pathotype track has an inner (pseudogene, truncation and protein length difference locations in white and missing genes in *red*) and outer (additional genes present in *green*) track. The three innermost tracks from outside to inside represent average GC content, sequence diversity and GC skew measured by a sliding window of 8 kb across the alignment. *Green* and *red peaks* on the GC content track represent two standard deviations either side of the mean (*grey line*). *Orange tips* on the sequence diversity track represent peaks that are more than two standard deviations above the mean. *Blue* and *red peaks* on the GC skew track are positive and negative values respectively

A summary of the genome sequencing results for the strains is given in Table 1. The GC content, GC-skew and sequence diversity measured for 8 kb sliding-windows over the alignment of all the strains are shown in Fig. 1. There is a clear correspondence between regions of high sequence diversity and low GC content. These regions also regularly coincide with detected genomic islands of differential gene content between the pathotypes, and detected regions of recombination. At the pathotype level, A* strains are the most diverse with an average genome polymorphism of 9.62 × 10^−4^ substitutions per site, compared to 6.41 × 10^−4^ and 5.27 × 10^−4^ substitutions per site for A and A^w^ respectively as measured by the Hamming distance of their aligned genomes.

**Table 1:** *X. citri* pv. *citri* strain isolation origin and sequencing information

Strain	Pathotype	Geographic origin	Isolation host	Year isolated	Reads	Contig number	Total (bp)	N50	Sequenced by	Mapped (bp)	% reads mapped
JJ10-1	A	Rodrigues Island	*C. aurantifolia*	1985	Single reads (100 bp)	335	5179910	54680	GATC	5027732	97.06
LG98	A	Bangladesh	*C. aurantifolia*	2006	Single reads (100 bp)	383	5164450	40179	GATC	5022236	97.25
JK143-11	A*	Thailand	Citrus sp.	1990	Single reads (100 bp)	384	5278157	54326	GATC	4993196	94.60
LB100-1	A	Seychelles	*C. sinensis x Poncirus trifoliata*	2005	Single reads (100 bp)	388	5263741	54697	GATC	5084444	96.59
JK4-1	A	China	Citrus sp.	1985	Single reads (100 bp)	401	5203097	42527	GATC	5065830	97.36
LG115	A^w^	India	Citrus sp.	2007	Single reads (100 bp)	408	5241689	48679	GATC	4921584	93.89
LG117	A	Bangladesh	Citrus sp.	2009	Single reads (100 bp)	429	5245224	53906	GATC	5046226	96.21
JM35-2	A*	Saudi Arabia	*C. aurantifolia*	1992	Single reads (100 bp)	441	5251255	54005	GATC	4956852	94.39
JS581	A*	Iran	*C. limetta*	1997	Single reads (100 bp)	450	5254826	54306	GATC	5019698	95.53
NCPPB 3607	A*	India	*C. aurantifolia*	1988	Single reads (100 bp)	520	5361783	28525	GATC	5003494	93.32
LH37-1	A	Senegal	*C. paradisi*	2010	Single reads (100 bp)	567	5405509	44606	GATC	5093867	94.23
NCPPB 3612	A	India	*C. aurantifolia*	1988	Single reads (100 bp)	585	5349385	52462	GATC	5082867	95.02
LE3-1	A*	Ethiopia	*C. aurantifolia*	2008	Single reads (100 bp)	1028	5300998	10596	GATC	4961125	93.59
JK48	A*	Saudi Arabia	*C. aurantifolia*	1988	Single reads (100 bp)	1054	5253806	9931	GATC	4917915	93.61
LG97	A	Bangladesh	Citrus sp.	2006	Single reads (100 bp)	1211	5252003	8460	GATC	4972934	94.69
LB302	A^w^	Florida	*C. aurantifolia*	2002	Single reads (100 bp)	1222	5239509	8709	GATC	4921146	93.92
LG102	A	Bangladesh	Citrus sp.	2006	Single reads (100 bp)	1232	5305763	8636	GATC	5015092	94.52
NCPPB 3610	A	India	*Poncirus trifoliata*	1988	Single reads (100 bp)	1247	5168073	7578	GATC	5027071	97.27
JK143-9	A*	Thailand	Citrus sp.	1990	Single reads (100 bp)	1314	5227724	7886	GATC	4939914	94.49
LE116-1	A	Mali	*C. aurantifolia*	2008	Single reads (100 bp)	1451	5355671	7770	GATC	5024296	93.81
NCPPB 3615	A*	India	*C. aurantifolia*	1989	Single reads (100 bp)	1546	5370437	6587	GATC	4938386	91.96
JS582	A*	Iran	*C. sinensis*	1997	Single reads (100 bp)	1622	5250292	6561	GATC	4892742	93.19
LD7-1	A	Mali	*C. aurantifolia*	2008	Single reads (100 bp)	1692	5340074	6225	GATC	5019242	93.99
LMG 9322	A*	Florida	*C. aurantifolia*	1986	Paired End (300/500 bp)	138	5195773	165596	Genoscope	5091334	97.99
FDC 1083	A	Brazil	*C. reticulata*	1980	Paired End (300/500 bp)	140	5219643	170725	Genoscope	5137607	98.43
FDC 217	A	Brazil	*C. sinensis*	2003	Paired End (300/500 bp)	146	5219970	148569	Genoscope	5138314	98.44
JJ238-10	A	Maldives Islands	*C. aurantifolia*	1987	Paired End (300/500 bp)	158	5262497	164415	Genoscope	5125732	97.40
JF90-8	A^w^	Oman	*C. aurantifolia*	1986	Paired End (300/500 bp)	164	5283250	120466	Genoscope	5120165	96.91
CFBP 2852	A	India	Citrus sp.	NA	Paired End (300/500 bp)	170	5274028	171317	Genoscope	5044668	95.65
X2003-3218	A^w^	Florida	Citrus sp.	2003	Paired End (300/500 bp)	171	5312286	110163	Genoscope	5052960	95.12
LD71a	A*	Cambodia	Citrus sp.	2007	Paired End (300/500 bp)	173	5282605	148601	Genoscope	4998550	94.62
JJ238-24	A*	Thailand	*C. aurantifolia*	1989	Paired End (300/500 bp)	173	5284713	164186	Genoscope	5049892	95.56
LC80	A	Mali	*C. reticulata x C. sinensis*	2006	Paired End (300/500 bp)	182	5232382	144093	Genoscope	5139614	98.23
JW160-1	A	Bangladesh	*C. aurantifolia*	2000	Paired End (300/500 bp)	202	5256256	155164	Genoscope	5055053	96.17
CFBP 2911	A*	Pakistan	Citrus sp.	1984	Paired End (300/500 bp)	202	5411197	163541	Genoscope	5111677	94.46
JF90-2	A*	Oman	*C. aurantifolia*	1986	Paired End (300/500 bp)	225	5257575	152298	Genoscope	4996177	95.03
NCPPB 3562	A	India	*C. limon*	1988	Paired End (300/500 bp)	230	5519974	148562	Genoscope	5112011	92.61
LE20-1	A*	Ethiopia	*C. aurantifolia*	2008	Paired End (300/500 bp)	462	5309008	138224	Genoscope	5047671	95.08
NCPPB 3608	A^w^	India	*C. aurantifolia*	1988	Paired End (300/500 bp)	517	5389095	114454	Genoscope	5061159	93.91
JS584	A*	Iran	Citrus sp.	1997	Paired End (300/500 bp)	575	5270551	144272	Genoscope	4961554	94.14
C40	A	Reunion Island	*C. sinensis*	1988	Single reads (100 bp) + Mate Pair (8 kb)	177	5241070	98653	Genoscope	5117602	97.64
JK2-10	A*	Saudi Arabia	*C. aurantifolia*	1988	Single reads (100 bp) + Mate Pair (8 kb)	318	5277475	67947	Genoscope	4959019	93.97

### Contig mapping

The results of the assembled contig-mapping to the strain IAPAR 306 reference are shown in Table 1. Between 91.96 and 98.43 % of the sequenced data was mapped to strain IAPAR 306 depending on the strain, with an average of 95.13 %. The regions from each strain that were not mapped to strain IAPAR 306 were not included in the comparative analysis because the exact relationships between homologous regions from these unmapped regions are difficult to define. While it was possible to map some contigs from each strain onto the two plasmids from IAPAR 306, there were no regions from the resulting alignment that were represented in all of the strain genomes or that were pathotype-specific.

### Recombination

Regions inferred to have undergone recombination across the *X. citri* pv. *citri* clade and the corresponding ranges of genes in strain IAPAR 306 are shown in Table 2 and Additional file 1. In total 21 regions were identified as likely being recombinant. Of these, seven events are inferred to have occurred on branches leading to the different pathotypes, five that are unique to the A^w^ pathotype and two that are unique to the A* pathotype. The five A^w^-specific recombination regions cover 80 genes in strain IAPAR 306 (Table 2 and Additional file 2), many of which were conferred A^w^-specific residues by the horizontal transfer. In general, the level of A^w^-specific residues is much higher in genes in these regions than in the rest of the genome with an average of 1.03 nonsynonymous SNPs per gene compared to a genome average in A^w^ strains of 0.0085 per gene (based on the A^w^ strain X2003-3218 annotation). Of the five events in the A^w^ pathotype, four of them appear to originate from *X. citri* pv. *bilvae* or a closely related bacterium. Of particular interest, an A*-specific recombination event and an A^w^-specific recombination event coincide within the *xopAD* gene, a type III effector. The effect of the different recombination events is that *xopAD* differs at many sites in a pathotype specific manner across all three pathotypes (Additional file 2).

**Table 2:** Regions of detected recombination in the whole genome alignment of all strains

Event	IAPAR 306 Coordinates	Alignment Coordinates	IAPAR 306 Start Gene	IAPAR 306 End Gene	Strain Presence	Putative Origin	Island
1	51959-65492	52850-66390	XAC0042	XAC0053	Aw	*X. citri pv.bilvae*	
2	214827-217043	219088-221270	XAC0174	XAC0176	JM35-2, JF90-2	Unknown	
3	243703-265596	248213-273302	XAC0198	XAC0217	Aw	*X. citri pv.bilvae*	Island1
4	1253042-1259543	1272074-1278440	XAC1101	XAC1107	A*	Unknown	Island2
5	1729051-1743732	1758692-1774868	XAC1497	XAC1509	Aw	Unknown	Island3
6	2931468-2932633	3000594-3001946	XAC2505	XAC2506	LE3-1, LE20-1	Unknown	
7	3256265-3256278	3344395-3344409	Intergenic	Intergenic	JJ10-1, C40	Unknown	
8	3525240-3525417	3615434-3615613	XAC3016	XAC3016	JS581, JS582, JS584, JK48, JK2-10	Unknown	
9	3538019-3542103	3629086-3634840	XAC3028	XAC3029	JM35-2	Unknown	
10	3839344-3842570	3940513-3945476	XAC3259	XAC3262	LB302, NCPPB 3218	Unknown	
11	3850723-3851839	3954394-3955620	XAC3269	XAC3269	JJ238-24, LD71a	Unknown	
12	3867931-3872268	3974477-3979351	XAC3288	XAC3293	NCPPB 3607	Unknown	
13	3868626-3877708	3975273-3986173	XAC3289	XAC3298	LG115	Unknown	
14	3872787-3872972	3980062-3980250	XAC3294	XAC3294	NCPPB 3607, JK143-9, JK143-11, LD71a, JJ238-24	Unknown	
15	3876659-3877403	3985023-3985867	XAC3298	XAC3298	LB302, X2003-3218	Unknown	
16	4257584-4467356	4370989-4598311	XAC3590	XAC3797	JJ238-10, LB100-1, CFBP 2852, JW160, NCPPB 3610, C40, JJ10-1, LMG 9322, JK4-1, FDC 217, IAPAR 306, FDC 1083, LC80, LG117	*X. citri pv.bilvae*	
17	4257584-4414966	4370989-4541201	XAC3590	XAC3740	LG98	*X. citri pv.bilvae*	
18	4364984-4366535	4490348-4491900	XAC3687	XAC3688	LG98	*X. citri pv.bilvae*	
19	4952518-4988729	5094257-5137776	XAC4204	XAC4227	Aw	*X. citri pv.bilvae*	Island4
20	4965286-4969165	5113255-5117325	XAC4213	XAC4213	A*	Unknown	Island4
21	5004702-5016321	5154088-5165863	XAC4239	XAC4250	Aw	*X. citri pv.bilvae*	

### Phylogeny

**Fig. 2:**
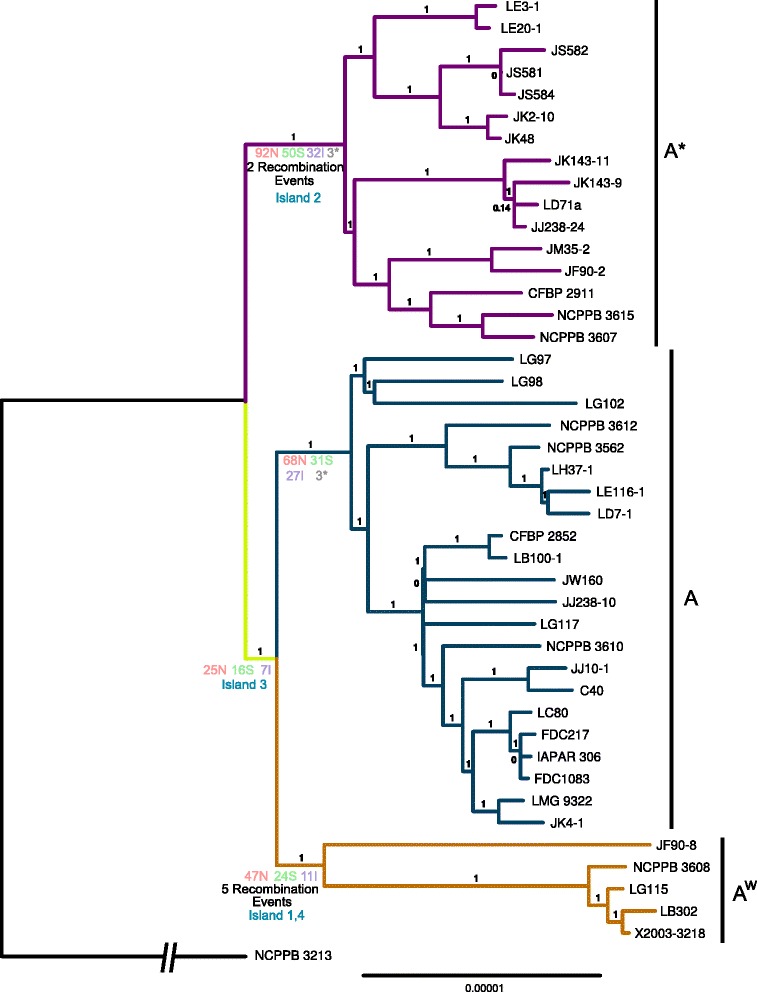
Phylogeny reconstructed from the whole genome alignment with removed regions of recombination. The pathotypes are colored *blue* (A), mauve (A*) and orange (A^w^) and the shared branch ancestral to A and A^w^ is colored *yelow*. The number of inferred nonsynonymous (N), synonymous (S), intergenic (I) and nonsense (*) SNPs, gene gains and losses and gene islands are marked along each branch. The outgroup branch has been shortened (indicated by the broken line) and is not to scale, to facilitate legibility of the figure

The phylogeny inferred from the whole genome alignment is shown in Fig. 2. Each pathotype in the phylogeny resolves into monophyletic groups, with the A and A^w^ pathotypes sharing a short branch after the divergence of A*. Consistent with previous Amplified Fragment Length Polymorphism and Multi Locus Variable number of tandem repeat Analysis (MLVA-31), A* strains formed robust subclusters in relation to their geographic origin [8, 10, 15]. Our phylogeny confirmed that the Indian subcontinent hosts unique A strains, including the ones referred to as DAPC 2 based on MLVA-31 [15] (NCPPB 3562, NCPPB 3612) and strains isolated in Bangladesh (LG97, LG98 and LG102), which form distinct subclusters within the A clade. In addition to strains originating from this region, the DAPC 2 lineage also included strains emerging in Mali and Senegal (LD7-1, LE116-1 and LH37-1) [16]. The strains JF90-8, LG115 and NCPPB 3608, previously designated as part of the A* pathotype [10], share a well supported branch with the other A^w^ strains, and are assigned as A^w^ in our work for this reason in conjunction with containing the gene island containing *avrGf1*, previously identified specifically in an A^w^ strain [11]. A second phylogeny constructed with recombination as part of the alignment and a reduced distribution of A strains similar to that of Zhang et al. [17] revealed the same pathotype branching structure as in this publication with the A* and A^w^ strains that share a branch (Additional file 3). Support for the pathotype-specific branches is very strong in both the recombination-containing and recombination-free phylogenies despite their differing topologies.

### Presence/absence analysis

We used orthologs identified by BLAST best reciprocal hits (BRH) (Additional file 4) in order to estimate the core-genome, pan-genome and number of singletons in our dataset, as well as to catalog gene presence and absence between pathotypes that are differentially present or absent in all strains of a pathotype. Genes related to IS elements and phages were excluded from the analysis due to their similarity, which makes orthology difficult to assign. We estimate that there are 2513 genes in the core-genome, 10,011 genes in the pan-genome and 2445 singletons spread across the 43 *X. citri pv. citri* strains. We identified four gene islands that are differentially present or absent between the pathotypes (Table 3, Fig. 1). Several genes previously identified as playing roles in pathogenicity or biofilm formation are found on these islands including the A^w^-specific *avrGf1* gene on Island 1 (previously identified as being differentially present between strain IAPAR 306 and A^w^ strain Xcaw12879 [12]), and *xrvA*, *mobL,* XAC1496, XAC1499 and XAC1509 on Island 3 that are absent from A*. Islands 2–4 all contain at least one gene that is usually plasmid-associated indicating their probable origin as plasmidic.

**Table 3:** Gene islands differentially present or absent across pathotypes

Island	A	A^w^	A*	Note	IAPAR 306 gene	X2003-3218 (A^w^) gene	LD71a (A*) gene	Gene name	Recombination
Island1	+	-	+	Absent in A^w^	XAC0211		XAC71A_110067	*gloA*	Event 3
	-	+	-	Unique to A^w^		XAC3218_110003		*avrGf1*	Event 3
	-	+	-	Unique to A^w^		XAC3218_110004			Event 3
Island2	+	+	-	Absent in A*	XAC1101	XAC3218_260032			Event 4
	-	-	+	Unique to A*			XAC71A_230028		Event 4
	-	-	+	Unique to A*			XAC71A_230029	*ccdB*	Event 4
	-	-	+	Unique to A*			XAC71A_230030		Event 4
	-	-	+	Unique to A*			XAC71A_240006		Event 4
	-	-	+	Unique to A*			XAC71A_240007		Event 4
	-	-	+	Unique to A*			XAC71A_240008		Event 4
	-	-	+	Unique to A*			XAC71A_240009		Event 4
Island3	+	+	-	Absent in A*	XAC1492	XAC3218_380022			
	+/−	+	-	Absent in A*		XAC3218_390001			
	+	+	-	Absent in A*	XAC1493	XAC3218_390002			
	+	+	-	Absent in A*	XAC1494	XAC3218_390003		*orf2*	
	+/−	+	-	Absent in A*		XAC3218_390004			
	+/−	+	-	Absent in A*		XAC3218_390005			
	+	+	-	Absent in A*	XAC1495	XAC3218_390006		*xrvA*	
	+/−	+	-	Absent in A*		XAC3218_390007			
	+	+	-	Absent in A*	XAC1496	XAC3218_390008			
	+	+	-	Absent in A*	XAC1497	XAC3218_390009			Event 5
	+/−	+	-	Absent in A*		XAC3218_390010			Event 5
	+/−	+	-	Absent in A*		XAC3218_390011			Event 5
	+	+	-	Absent in A*	XAC1499	XAC3218_390012			Event 5
	+	+	-	Absent in A*	XAC1500	XAC3218_390014			Event 5
	+/−	+	-	Absent in A*		XAC3218_390015			Event 5
	+/−	+	-	Absent in A*		XAC3218_390016			Event 5
	+/−	+	-	Absent in A*		XAC3218_390017			Event 5
	+/−	+	-	Absent in A*		XAC3218_390018			Event 5
	+	+	-	Absent in A*	XAC1501	XAC3218_400002			Event 5
	+	+	-	Absent in A*	XAC1502	XAC3218_400003			Event 5
	+	+	-	Absent in A*	XAC1503	XAC3218_400004			Event 5
	+	+	-	Absent in A*	XAC1506	XAC3218_400005			Event 5
	+/−	+	-	Absent in A*		XAC3218_400006			Event 5
	+/−	+	-	Absent in A*		XAC3218_400007			Event 5
	+	+	-	Absent in A*	XAC1507	XAC3218_400008		*mobL*	Event 5
	+	+/−	-	Absent in A*	XAC1508	XAC3218_400010			Event 5
	+	+/−	-	Absent in A*	XAC1509	XAC3218_400011			Event 5
Island4	+	-	+	Absent in A^w^	XAC4205		XAC71A_950084		Event 18
	+	-	+/−	Absent in A^w^	XAC4206				Event 18
	+	-	+	Absent in A^w^	XAC4209		XAC71A_960001	*cvaB*	Event 18
	-	ψ	-	Unique to A^w^		XAC3218_960285			Event 18
	-	ψ	-	Unique to A^w^		XAC3218_960286			Event 18
	-	+	-	Unique to A^w^		XAC3218_970001			Event 18
	-	ψ	-	Unique to A^w^		XAC3218_970002			Event 18
	-	ψ	-	Unique to A^w^		XAC3218_970003			Event 18

+ present in all strains of a pathotype; − absent in all strains of a pathotype; +/− present in some strains of a pathotype; ψ putative pseudogene

All of the islands except the A^w^-specific Island 1 containing *avrGf1* coincide with island locations identified in strain IAPAR 306 with IslandViewer [18]. Island 3 partially coincides with an A^w^-specific recombination event of unknown origin, while Island 1 and Island 4 fully overlap A^w^-specific recombination events originating from *X. citri* pv. *bilvae*. Island 2 fully overlaps an A*-specific recombination region of unknown origin. Apart from these islands and several likely pseudogene fragments (see below), we did not identify any genes that were exclusively present or absent in a given pathotype.

### Ancestral character estimation

Using ancestral character estimation, a total of 426 SNPs were mapped onto the four branches leading to the three pathotypes, of which 350 are genic and 76 are intergenic based on the annotation of strain IAPAR 306 (Fig. 1 and Additional file 5). There are 220 nonsynonymous mutations and 124 synonymous mutations as well as nonsense mutations in three A and three A* genes.

Among the nonsynonymous SNPs, there are many in genes with previously identified putative roles related to pathogenicity or biofilm formation in various *Xanthomonas* species. Specific to the A pathotype, these include genes related to secretion systems or effectors: *avrBs2, xopN*, *xopL*, *hrpE*, *hrcU, lamA* [18–23]; genes related to EPS production and biofilm formation or regulated by DSF: *rpfA*, *rpfB, gumL*, *gumD, cyoC, fecA* [11, 24–35]; and iron transport: *fhuA* [11]. On the A* branch are nonsynonymous SNP-containing genes related to secretion systems or effectors: *hrpB5, hrpXct, xopX, xopK, xopL, xcsG, xcsN, secE* [18–22, 34]; biofilm formation: *tsr* and *gumL* [27–29, 32, 35, 36]*;* and *iron transport: fhuA* [11]. Aw-specific nonsynonymous-SNP containing genes include genes related to secretion systems or effectors: *xopP, xopL, hpaB* [20, 22, 23]; genes related to EPS production and biofilm formation: *gumM, gumD, tsr* [27–29, 35–37]*,* organic hydroperoxide resistance gene *osmC* [36], a xylanase *xynB* [38] and XAC4203, a mutant of which is biofilm defective [35]. Finally, on the shared A/A^w^ branch, there are nonsynonymous mutations in *rpfB,* and the adhesion-associated protein *yapH* [39, 40].

### Pseudogenes, frameshifts and truncations

Pseudogenes, frameshifts and truncations were identified by the complementary approaches of the protein length analysis, ancestral character estimation and the presence/absence analysis (Table 4). There are eight identified putative pseudogenes in pathotype A strains, 15 in A* strains and five in A^w^ strains. Several of the putative pseudogenes are reported to have roles in defence: the catalase *catB* [41, 42] and permease *rarD* [43] involved in drug resistance in the A strains and *yojM* [44], a superoxide dismutase-like gene in the A^w^ strains. There are also putative pseudogenes in the A* strains that are involved in pathogenicity or biofilm formation: *rpfB* [24, 25, 29–31]*,* a regulator of pathogenicity factors, a GGDEF domain-containing protein-encoding gene [29, 36] and the type III effectors *xopN* and *xopL* [20, 22].

**Table 4:** Pathotype-specific putative pseudogenes, genic frameshifts and truncations

Pathotype	Gene	Description	Native coordinates	Alignment coordinates	Gene fragment 1	Gene fragment 2	Note
A		methyltransferase domain	60851-61836	61748-62734	XANAC_0061	XAC0050	Putative ψ
	LacZ	beta-galactosidase	838208-841101	850951-853845	XAC0707	XAC0708	Putative ψ
		putative secreted protein	986002-986826	999771-1000595	XAC0825	XANAC_0965	Putative ψ
	oppD/yliA	ABC transporter	1021079-1022728	1034920-1036570	XAC0859	XAC0860	Putative ψ
	araJ	MFS transporter	3326112-3327329	3414846-3416063	XAC2837	XAC2836	Putative ψ
	rarD	Permease – chloramphenicol resistence	4693056-4693966	4830382-4831292	XAC4000	XAC4001	Putative ψ
	catB	catalase	4719198-4720710	4856716-4858239	XAC4029	XAC4030	Putative ψ
A*		PbsX Transcription Factor	614013-614472	630726-631530	XAC71A_130207	XAC71A_130208	Putative ψ
		acyl-CoA synthetase	1041072-1042552	1063009-1064489	XAC71A_170122	XAC71A_170123	Putative ψ
			1622056-1625651	1640494-1644089	XAC71A_280103	XAC71A_280104	Putative ψ
			1860274-1860803	1904352-1904881	XAC71A_310052	XAC71A_310053	Putative ψ
	rpfB	acyl-CoA synthetase	2187629-2189311	2300143-2301825	XAC71A_390075	XAC71A_390076	Putative ψ
			2269033-2269866	2382037-2382870	XAC71A_410007	XAC71A_410008	Putative ψ
		GGDEF family protein	2286218-2289170	2399383-2402335	XAC71A_410020	XAC71A_410021	Putative ψ
	comA	competence protein	2447016-2449565	2572127-2574695	XAC71A_450029	XAC71A_450030	Putative ψ
	XACSR11	carboxypeptidase	3101427-3102721	3299904-3301198	XAC71A_660002	XAC71A_660003	Putative ψ
	xopN	type III effector	3293500-3295557	3508783-3510840	XAC71A_730134		Putative ψ, truncated 3′
	yagT	putative oxidoreductase, 2Fe-2S subunit	3420879-3421529	3641125-3641775	XAC71A_740038	XAC71A_740039	Putative ψ
	yodB	Cytochrome B561	3563369-3564046	3785148-3785825	XAC71A_750009		frameshift, longer 3′
	xopL	type III effector	3641485-3643372	3864216-3866109	XAC71A_760048	XAC71A_760049	Putative ψ
	rimK	Ribosomal protein S6 modification protein	3847131-3848050	4111446-4112365	XAC71A_840004	XAC71A_840005	Putative ψ
			3998076-3997795	4263201-4262920	XAC71A_880069		Putative ψ, truncated 5′
A^w^			59860-61116	60605-61861	XAC3218_20024	XAC3218_20025	Putative ψ
	adh	alcohol dehydrogenase	247107-247385	256015-256293	XAC3218_100056		Putative ψ, truncated 3′
	yojM	superoxide dismutase-like	256565-257695	265546-267635	XAC3218_110001	XAC3218_110002	Putative ψ
			3010085-3008397	3123577-3121889	XAC3218_630012		longer
		LysR Transcription Factor	4453733-4452393	4671211-4669871	XAC3218_910186		longer
		exported	4957802-4960197	5228371-5230773	XAC3218_960285	XAC3218_960286	Putative ψ
			4962702-4964291	5233406-5234995	XAC3218_970002	XAC3218_970003	Putative ψ
	ttcA	2-thiocytidine biosynthesis protein TtcA	4988408-4989507	5261994-5263093	XAC3218_990014	XAC3218_990015	Putative ψ
	xylB	xylulose B	5015663-5013780	5290774-5288891	XAC3218_990034		longer

### COG enrichment analysis

COG categories enriched in the nonsynonymous SNP, missing and recombination gene sets are shown in Additional file 6. The majority of COGs enriched in the gene sets appear to be involved in the transport and metabolism of various compounds. The gene set with the most enriched COGs are the missing genes from A*, which has 21 enriched COGs. Both A* and A^w^missing gene sets are enriched for COGs involved in defense mechanisms, while all the missing genes that are COG-enriched in A-strains are involved in compound transport and metabolism. The most significantly enriched category in A* missing genes is transcription, involving three different transcriptional regulator genes, two of which reside in Island 3. Amongst the nonsynonymous SNP gene sets, the A/A^w^ branch has the most enriched COGs that are mostly involved in transport and metabolism (11 of 14 genes). Despite having the most branch-specific nonsynonymous SNPs, the only enriched category for A*-specific SNP genes is for genes that have not been assigned a COG. In pathotype A, the most significantly enriched COG in the SNP gene set is involved in defense mechanisms, and includes the transport protein gene acrD and the multidrug efflux protein genes *mexB* and *smeB.* Energy production and conversion processes are also over-represented by *avrBs2* and *glpQ,* genes that both contain a glycerophosphoinositol phosphodiesterase (GDE) domain [18]. The shared A/A^w^ branch, has several enriched COGs. In the recombinant regions in A^w^, COGs for intracellular trafficking, secretion, and vesicular transport and inorganic ion transport and metabolism are enriched due to the presence of the *tatA*/*tatB* translocation system and two modular superoxide dismutase genes respectively.

## Discussion

The focus of our study is to trace the evolutionary events that led to the emergence of three pathotypes of *X. citri* pv. *citri* that may explain differences in host range and virulence between them. As each pathotype is defined by its host-range or HR on different hosts, we searched for the genomic differences that appear to be entirely present or entirely absent from a pathotype ranging from the level of single base changes to multi-gene islands (Table 3 and Fig. 1). These events were placed into their evolutionary context by a phylogenetic reconstruction of all strains from non-recombinant regions of the multiple genome alignment.

Detection and removal of recombinant sequences allows the construction of a phylogeny that theoretically represents the true relationship of the vertically inherited genome portions of the strains (Fig. 2). The phylogeny shows monophyletic groups for each pathotype and suggests that the A^w^ and A pathotypes share a branch to the exclusion of the A* pathotype. The topology agrees with the overall structure of previous AFLP [10] and MLVA [15] phylogenies of the *X. citri* pv. *citri* group. Some strains studied by Escalon et al. [10] assigned to A* (JF90-8, LG115 and NCPPB 3608) are probably in fact A^w^ strains based on the molecular phylogeny presented here and consistent with the hypersensitive response in grapefruit and sweet orange due to the presence of *avrGf1* [10, 11]. A recent publication [17] reconstructed a tree with a different branching order of the three pathotypes to that found in our reconstruction. In the published phylogeny, the A^w^ and A* pathotypes clade together to the exclusion of A strains. By reconstructing the relationship between the pathotypes both with (Additional file 3) and without regions of recombination (Fig. 2), we show that the topology from this publication is influenced by regions of recombination which were not removed before the reconstruction of the phylogeny, making it most likely incorrect. Because regions of recombination violate the core assumption of common evolutionary history for all of the sites in an alignment, it is important to control for their presence when reconstructing phylogenies [45]. Additionally, as the tests of positive selection used in the publication rely on a correct tree topology, the major result of this publication – that positive selection is the main driving force behind the evolution of citrus canker-causing *Xanthomonas* species – is therefore uncertain.

The only widely distributed common host species for all of the *X. citri* pv. *citri* pathotypes and the outgroup *X. citri* pv. *bilvae* is Key lime (*Citrus aurantifolia)*. Based on our phylogeny, any differences in pathogenicity and host range between the A and A^w^ pathotypes should be isolated to events occurring on either or both of the branches leading to the two groups. Apart from a hypersensitive response in grapefruit and sweet orange [10, 11], which can largely be explained by the presence of *avrGf1* [11], the host range and specificity of A^w^ is narrow and similar to that of A* [10], suggesting that the larger host range of the A group has developed along the branch leading to the A pathotype alone and that the ancestor of the A and A^w^ pathotypes had a host range and virulence similar to the A^w^ and A* groups. Less parsimonious scenarios cannot be ruled out, e.g. that A^w^ (or indeed both A^w^ and A*) evolved a restricted host range from an ancestor that had a broad host range.

The four gene island regions that show differential presence and absense of genes across the pathotypes are all coincident with detected regions of pathotype-specific recombination, which suggests that these islands have been gained in certain pathotypes rather than lost in pathotypes where they are absent. In addition, the fact that all but the island containing the *avrGf1* gene contain genes that are normally associated with plasmids (*ccdB*, *mobL*, *cvaB*) indicates a likely xenologous plasmid origin. Interestingly, gene islands were also regions that contained missing genes between pathotypes. Except for putative pseudogenes (Table 4) all of the genes whose presence or absence is different between pathotypes are associated with regions of recombination or islands that are mostly of probable plasmid origin.

Although it might be expected that genes contained on these islands could define host range, there are no genes that are present or absent exclusively in A strains. Genes differentially present between the A and A^w^ pathotypes are found on Island 1 and Island 4 (Table 3), including *avrGf1*. Although *avrGf1* deletion does not confer A-like host range to A^w^ strains, indicating that the presence or absence of other factors is also necessary to explain differing host ranges [10, 11], expression of *avrGf1* or its homolog *avrGf2* in A strains does illicit a HR in grapefruit [12]. Furthermore, in the case of both of these islands, A and A* strains share the same presence and absence patterns, indicating that they are likely not host range determining. Despite containing genes involved in virulence and biofilm formation (*xrvA*, *mobL*, XAC1496, XAC1499 and XAC1509) [34, 35, 46, 47], Island 3 is also unlikely to be a key factor in host range because it is present in A and A^w^ strains. In any case, the presence and absence differences restricted to gene islands that tend to coincide with zones of detected recombination outline the large role of horizontal transfer, plasmid insertion and recombination in the genomic evolution of *X. citri* pv. *citri* pathotypes.

In contrast to a recent analysis of *X. citri pv. citri* strains [17] we did not identify any additional pathotype-specific genes in the A strains. To validate our results, we checked the genes that differ between that analysis and our results (Additional file 7). Overall the majority of differences are due to fragments of existing genes that appear to have been split or pseudogenized, genes that are in fact found in other pathotypes, or genes found restricted to a pathotype, but not in all member strains.

A gene region identified in A-strains [17] that is involved in LPS biosynthesis is a gene island that corresponds to a region of detected recombination (event 16) in our data. However, the distribution is restricted to 14 of the 22 A-strains meaning that while it may have a large effect on biofilm formation and O-antigen composition in these strains as demonstrated by SDS-polyacrilamide gel electrophoresis [17], it is not the key factor to different host ranges between the pathotypes as a whole.

As well as gene islands and regions of recombination, host range differences may also be caused by pseudogenization. However, caution is needed when inferring pseudogenes from frameshifts or stop codons, as ribosomal frameshifting and transcriptional realignment can lead to fully-functional transcripts and proteins despite apparent non-functional coding sequences [48].

In the A pathotype, the only putative pseudogene known to be involved in virulence, is *catB,* a putative monofunctional catalase that may be involved in the detoxification of reactive oxygen species produced by plants during their defense processes [41, 42]. An EZ::TN transposon insertion in *catB* in *X. campestris pv. campestris* reduces virulence of the bacterium on its host plant [41]. Supporting its pseudogenic nature in strain IAPAR 306, Tondo et al. [42] could not detect *catB product* in *X. citri* pv. *citri* using RT-PCR.

Although several potential pseudogenes associated with infection are found in A* strains, e.g. *rpfB, xopL, xopN* and a GGDEF family protein (Table 4), these are unlikely to be the root cause of host range differences between the pathotypes given the phylogeny and their distribution across the different pathotypes, however they may still cause differences in virulence between the pathotypes.

The pathotypes also differ by mutations and recombination events that have overwritten homologous native sequence. A large number of the molecular differences between A and A^w^ strains comes from the five A^w^-specific recombination events (Fig. 1, Table 2, Additional file 2) spanning 80 genes in strain IAPAR 306 (Additional file 1). Many of the genes contain one or more A^w^-specific nonsynonymous substitution and in several cases also contain frameshifts, truncations and indels that are specific to A^w^ strains. Interestingly, four out of the five detected pathotype-specific recombination events in A^w^ appear to originate from *X. citri* pv. *bilvae,* as well as three other detected events*,* indicating a physical interaction must have occurred at some time in the past, most likely in India and probably on the common lime host. *X. citri* pv. *bilvae* has thus played a large role in the genomic evolution of the *X. citri* complex, specifically in the A^w^ pathotype.

Notably, one of the regions of A^w^ recombination contains *xopAD,* a type III effector that has previously been noted by Escalon et al. [10], as being the likely subject of recombination. Interestingly, the region containing *xopAD* appears to have undergone pathotype-specific recombination in both A^w^ and A* pathotypes, meaning that there are pathotype-specific versions in all three pathotypes. The A* and A^w^ versions contain multiple pathotype-specific amino acid residues (69 and 59 respectively). Furthermore, in the A^w^ strains (excluding JF90-8) a transposase from an ISXac5 element interrupts the *xopAD* gene at the 3′ end [10]. Jalan et al. [11] found that *xopAD* is upregulated in Xcaw12879 compared to strain IAPAR 306, suggesting that the IS interruption may not have completely pseudogenized this gene in A^w^, although the truncation could potentially affect its function where it is present. Deletion of *xopAD* in A strains does not appear to affect its pathogenicity on different citrus hosts in strain IAPAR 306 [10], but as it is a Type III Effector and has unique versions of the gene in each pathotype it remains a good candidate for future study of pathotype-specific host range.

**Fig. 3:**
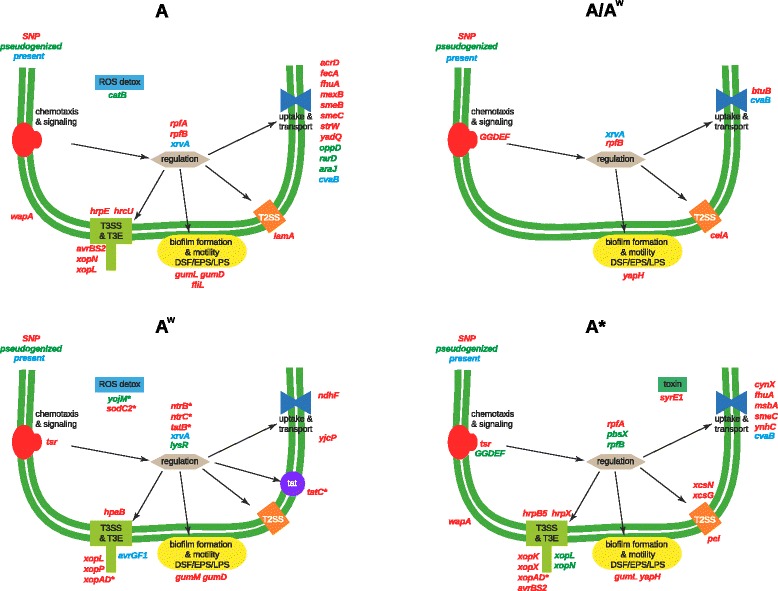
Schematic representation of a selection of genes involved in pathogenicity or fitness functions that are involved in pathotype specific events. Events are SNPs (in *red*), pseudogenization (in *green*), or presence in genomic islands (in *blue*). Events marked with an asterisk are present in regions of detected recombination

Figure 3 summarizes our results focusing on genes coding for factors that differ between pathotypes in terms of SNPs, pseudogeneization or presence/absence and are potentially involved in host specialization, i.e. related to functions that allow virulence on a plant species but not on another or that differentiate strains that are or are not pathogenic on a given species. For example a bacterial strain would not be pathogenic if: it cannot survive on the plant surface (involving extracellular polysaccharides, quorum sensing etc.); it cannot detect and/or swim to openings (sensors, flagellar system etc.); it cannot inhibit and/or evade plant defenses (Type III effectors (T3E), pathogen/microbe associated molecular patterns, detoxification, iron mobilization, molecular targets of defense mechanisms etc.); it cannot cause symptoms to disrupt plant tissues to liberate nutriments and/or exit (T3E, enzymes, toxins etc.); it cannot uptake and/or use nutriments (CUT system, transport, enzymes etc.). More generally deficient sensing, signal transduction or regulation may be involved in the inability to cause disease.

In our dataset, the low level of polymorphism along any given branch of the tree (the majority of inferred mutated genes contain only a single polymorphism specific to the pathotype branch) does not provide enough power to estimate selective constraints accurately in a pathotype-specific manner [49]. Several genes contain more than one nonsynonymous mutation per pathotype or across pathotypes, making them interesting candidates as potential targets of selection.

Genes that are already known to play a role in pathogenicity are also prime candidates for the evolution of host range, especially genes that have differences in an A-specific manner. These include nonsynonymous SNP-containing genes involved in secretion systems, *hrpE*, *hrcU*, *avrBS2*, *xopN, xopL* and *lamA*; genes involved in regulation of pathogenicity factors, *rpfA* and *rpfB*; genes involved in biofilm formation and motility related to diffusable signal factor (DSF), EPS and LPS, *gumL*, *gumD* and *fliL* as well as an array of genes involved in uptake and transport of various compounds including the iron transporters *fecA* and *fhuA*. The *rpf* (regulation of pathogenicity factors) genes are involved in cell-cell signalling via diffusable signal factor and in the regulation of the synthesis, polymerization and secretion of extracellular enzymes and polysaccharides (xanthan) [24–29]. Notably, *rpfB* has two A-specific nonsynonymous substitutions as well as an A/A^w^ nonsynonymous substitution and is a putative pseudogene in the A* strains. Mutation of *rpfB* leads to loss or severe reduction of DSF activity (< 10 % of wild type) in *X. campestris* pv. *campestris* [30, 31]. Furthermore, there are at least six A-specific nonsynonymous SNP-containing genes (*cyoC, gumL, gumD, mexB, fecA* and *xopL*) that are reportedly regulated by DSF in *X. campestris* pv*. campestris* and *X. oryzae* pv*. oryzae* [28, 32, 36]. As they are important in cell-cell signalling as well as the regulation of pathogenicity factors and biofilm formation (i.e. they exist at the crossroads of sensing and gene expression), the *rpf* genes and their regulation targets that contain SNPs are good candidate genes to explain the differentiation of the pathotypes in terms of their virulence and host range.

The significant enrichment of mutated, missing (including putative pseudogenes) or recombined genes in functional categories may also indicate possible selection acting on various bacterial systems. Interestingly in A strains, the enriched missing gene functions are all involved in the transport and metabolism of various compounds (Additional file 6). While A^w^ and A* missing genes are also enriched for transport and metabolism functions, both pathotypes are also depleted for genes involved in defense mechanisms (XAC1388 and *cvaB* respectively), and A* is depleted for genes involved in transcription (XAC1493, XAC0524, XAC1499), posttranslational modification, protein turnover, chaperones (XAC1101), energy production and conversion (*yagT*), replication, recombination and repair (*mobL*) and cell wall/membrane/envelope biogenesis (*rimK*). Furthermore the A strains are enriched for genes containing SNPs that are involved in defense mechanisms (*acrD*, *mexB* and *smeB*), energy production and conversion (*glpQ* and *avrBs2*) and intracellular trafficking, secretion, and vesicular transport (*acrD* and *smeB*), while neither A^w^ nor A* are enriched for any functions for genes containing SNPs. Multidrug efflux systems may be important in the pathogenicity of the bacteria, potentially by protecting the bacteria against plant antimicrobials released as a defense response [50, 51]. Indeed, knockout of the m*exB* gene along with associated *mexA* and *oprM* genes in *Pseudomonas syringae* causes a major reduction in the bacterial populations *in planta* [50]. Furthermore, it has been suggested that multidrug efflux pumps may play a role in exporting quorum sensing molecules out of the cell as well as flagellar motility which is associated with biofilm formation [51].

## Conclusion

In this work we present a comparative genomics analysis using one previously sequenced strain and the draft genome sequences of 42 strains of *X. citri* pv. *citri*, and one *X. citri* pv. *bilvae* strain as an outgroup. Each of the three pathotypes of *X. citri* pv. *citri* are monophyletic, and we found multiple differences between the genomes of three pathotypes of *X. citri* pv. *citri* ranging from differences in gene content, putative pseudogenization, and nonsynonymous mutations in several genes known to be involved in various aspects of pathogenicity*.* We find that there is extremely little variation in gene-content at the pathotype level: apart from potential pseudogenes all of the detected differences in gene content are linked to gene islands and regions of recombination, indicating that HGT and recombination have been major factors in the gene content evolution of *X. citri* pv. *citri* pathotypes in terms of both the gain and the loss of gene content and mutations. Few content differences exist between A and A^w^ strains despite A^w^ strains having a narrow host range similar to that of A*.

Our analysis of recombination detected multiple recombination events across the genomes, with seven detected events that are specifically present only in all members of a pathotype. Five of the pathotype-specific events are in A^w^ strains, and two are in A*. Four of the A^w^-specific recombination events are inferred to originate from a bacterium related to *X. citri* pv. *bilvae* due to high sequence similarity, suggesting an interaction between the A^w^ strain ancestor and this *X. citri* pv. *bilvae-*like strain, probably in a lime host. Interestingly, our analyses demonstrated that due to two recombination events, the *xopAD* gene displays pathotype-specific versions in all three pathotypes, making it a good candidate for further study of the host range or pathogenicity differences between the pathotypes. There are also several other notable differences of potential importance between the pathotypes that may explain differences in host range and pathogenicity, notably *rpfA* and *rpfB* genes that are involved in regulating pathogenicity factors and biofilm formation through DSF production. These genes and several of their targets contain multiple SNPs along different pathotype branches. We also identify several COGs that are either significantly enriched or depleted for the individual pathotypes and may suggest selection acting on certain functions in the pathotypes.

Overall, our study provides insights into the genomic evolution of the pathotypes of *X. citri pv. citri* and provides candidates for further study into their different host ranges and virulence.

## Methods

### Sequencing and assembly

Xanthomonas strains (Table 1) were stored at −80 °C as freeze-dried cultures and cultivated on YPGA (yeast extract 7 g/L, glucose 7 g/L, peptone 7 g/L, agar 18 g/L, pH 7.2), as described previously [14]. Genomic DNA was isolated using Promega Wizard Genomic DNA Purification kit (Promega, Charbonnières, France) according to the manufacturer instruction. DNA quantity and quality were assessed by nanodrop and gel electrophoresis. Illumina sequencing was performed by GATC (23 strains with single read length of 100 bp) and Genoscope (17 strains paired end reads of 300/500 bp and three strains with combined single reads of length 100 and 8 kb mate-pair reads). Assembly was performed by Genoscope and in-house for the GATC strains using the Velvet assembler [52].

Gaps in *xop* gene sequences were closed using PCR. All PCR runs were performed with a GeneAmp PCR system 9700 thermocycler (Applied Biosystems, Saint Aubin, France). PCR was performed in 20-μL reaction mixtures containing 1 × Gotaq® green buffer (Promega), 1.5 mM MgCl2, 200 mM of each deoxynucleoside triphosphate (dNTP), 0.3 mM of each primer, 2 ng of template genomic DNA and 0.8 U of GoTaq® Polymerase. The amplification program consisted of 35 cycles of denaturation at 95 °C for 45 s, annealing at 55 °C for 45 s and extension at 72 °C for 0.5–2 min, depending on the length of the PCR product (1 kb/min) (primers used for contigs assembly will be provided upon request). All PCR products to be sequenced (Sanger technology) were sent to Beckman Coulter Genomics (Stansted, Essex, UK). Sequence assembly and alignments were performed using Geneious software v5.5.6 [53].

### Contig mapping

The assembled contigs for the 43 strains were mapped onto the complete reference genome of *X. citri* pv. *citri* strain IAPAR 306 [54] using BLAST [55] with manual curation. Contigs of less than 200 bp were removed. The contigs were initially filtered for plasmid sequences with BLAST [55] against a database of whole plasmid sequences from *X. citri* pv. *citri* strain IAPAR 306, *X. euvesicatoria* strain 85–10 [19], *Xanthomonas fuscans* subsp. *fuscans* [56] and the plasmids from a further two *X. citri* pv. *citri* strains, C40 and JK2-10. To map to the reference contigs were required to be at least 90 % identical to the reference over at least 20 % of their length and be the top hit to a given region. In cases of duplicate contigs, the top scoring hit was mapped to the reference. The mapped genomes for all the strains were aligned using Mugsy [57], and the resulting aligned blocks were ordered according to strain IAPAR 306. The same mapping process was attempted using the two plasmids of strain IAPAR 306 as references for the remaining unmapped contigs after the initial genome mapping stage. The genome alignment data were deposited in the Dryad online repository (http://dx.doi.org/10.5061/dryad.8t53k).

### Quality checking

Raw reads were mapped to the assembly of each strain using Bowtie2 [58] before compiling information on each position using the Samtools suite [59]. VCFtools [60] was used to convert the output from Samtools into readable plaintext. Positions with a quality score of < 40 were cross-referenced to the multiple genome alignment, corrected where possible, or changed to an “N”.

### GC content, GC skew and diversity

A Python script was used to calculate GC content, GC skew and sequence diversity. Sliding windows of 8 kb were passed along the alignment, and the average values were plotted using Circos [61]. Sequence diversity was measured using Hamming distance [62].

### Recombination analysis

RDP v4.16 [63] was used to detect regions that have undergone recombination. The alignment was reduced to a length of 56,705 by extracting SNP columns from the whole genome alignment blocks. IAPAR 306 was used to order the SNPs so only SNPs (including gaps) from alignment blocks that were present in strain IAPAR 306 were included in the SNP alignment. The SNPs from regions not present in strain IAPAR 306 were examined but no pathotype-differentiating SNPs between A* and A^w^ were found. Regions identified as likely recombination events (Table 2, Additional file 1) were subsequently removed from the alignment to mitigate the confounding effects of recombination on other analyses. Regions of detected recombination were checked for spurious signals due to poor alignment, mapping, contig joins or low quality sequence.

### Phylogeny

A phylogenetic tree was reconstructed from the full genome alignment (Fig. 2). Detected regions of recombination as well as gap columns and 20 bp either side were removed as these regions are often at the edges of contigs and are more likely to contain sequencing errors or regions of poor alignment. Model selection was performed with jModeltest 2.1.7 [64], and the SYM model chosen using the Bayesian Information Criterion. The phylogeny was reconstructed using PhyML [65] with chi-square branch support. A phylogeny from an alignment containing regions of recombination and a reduced distribution of A strains was also constructed to compare with a recently published phylogeny of *X. citri pv. citri* strains [17] (Additional file 3). The A strains used were those containing recombination Event 16, which was reported to be present in all A strains in the published dataset. To match the phylogeny in the recent publication, recombination-containing phylogeny was constructed using PhyML under the GTR model, with chi-square branch support values. The trees were visualized with Figtree v1.4.1 [66]. Both phylogenies and alignments were deposited in the Dryad online repository (http://dx.doi.org/10.5061/dryad.8t53k).

### Ancestral character estimation

From an alignment of 46,072 SNP positions, strain-specific SNPs were removed to decrease the time for ancestral character estimation concentrating specifically on certain internal branches of the phylogeny. The resulting SNP alignment was 2954 bases long. To check that removal of these sites doesn’t alter the branching orders, a phylogeny was constructed with MrBayes v3.2.1 [67, 68]. Two Markov chains were run for 10,000,000 generations using “reversible jump MCMC” to sample across different substitution schemes, with sampling every 500 generations. The sample parameters and trees were summarized and the first 10 % were removed as burn-in. Tracer [69] was used to check for convergence. The resulting SNP tree (Additional file 8) returned the same pathotype branching topology as the full phylogeny based on the genome alignment.

Using the constructed SNP phylogeny, each node in the alignment was constrained and two runs with four Markov chains were performed simultaneously for 5,000,000 generations and sampled every 500 generations. The sample parameters and trees were summarized with removal of the first 10 % as burn-in and tracer was used to check for convergence for each analysis. Mutations were placed onto specific branches of the phylogeny if different nucleotides were inferred to be at the same alignment position at adjoining nodes, and the average difference in probability for the given residues at the two nodes was greater than 0.5.

### Presence/absence analysis

Gene annotations were performed using an automated pipeline implemented by MaGe [70, 71]. Predicted proteins were compiled into a database and an all-against-all BLASTP was performed. Proteins associated with IS elements, and phage-related proteins were flagged and removed. Best reciprocal hits (BRH) to *Xanthomonas citri* strain IAPAR 306 were assigned from each of the other strains. Proteins from other genomes that were unassigned in the first round BRHs were then used as queries against the remaining genomes until all genomes had been examined. For each group of BRH assignments (Additional file 4), the distribution of the proteins in A, A* and A^w^ were examined to find those that were completely present in at least one of the pathotypes while being completely absent in at least one of the others. Split genes were identified by comparing each protein against the entire protein set from all strains, and finding instances where it hit adjacent genes in one of the other strains that had no homology among themselves. If a gene missing from a strain coincided with a contig break, it was considered as an unknown requiring manual examination in any cases that conformed to a potential pathotype-specific pattern. We identified groups of adjacent genes differentially present or absent from whole pathotypes as pathotype-specific gene islands. The core-genome, pan-genome and number of singletons were estimated from the BRH table. Notably, these estimates do not include IS elements, because the orthologous relationship for these very similar genes is difficult to define. It should also be noted that given the unfinished nature of the genome sequences, it is not possible to definitively infer the absence of a gene, which may affect both the presence/absence analysis and the core-genome and pan-genome estimates. However given the high coverage of NGS data, it is expected that most if not all non-repetitive regions should be present in the assembled contigs.

### Length analysis

To find genes that differed between pathotypes due to indels, splitting of genes or pseudogenization, an analysis of the lengths of annotated proteins from the BRH table (Additional file 4) was performed. The average length, standard deviation and coefficient of variation was measured for a given identified ortholog in each pathotype. Protein alignments were created and manually examined for candidates where the average length varied between pathotypes with a within-pathotype coefficient of variation of less than 0.05 (Additional file 9).

### COG enrichment analysis

From the classification in 1837 COGs annotated by MaGe for IAPAR 306, an analysis of COG enrichment was performed on the pathotype-specific presence/absence data, recombinant regions and genic nonsynonymous SNPs. COGs annotated in *X. citri* strain IAPAR 306 were used for A-specific and A/A^w^ shared branch nonsynonymous SNPs and also for genes that were missing from the A* and A^w^ pathotypes (Additional file 6). The missing gene sets include putative split or pseudogenes and truncated genes. For A*-specific SNPs and recombination, COGs and genes from strain LD71a were used, and for A^w^-specific SNPs and recombination and A-specific missing genes, COGs and genes from strain X2003-3218 were used. For the genic SNP sets, each gene was considered once even if it contains multiple nonsynonymous mutations. For each COG category in the gene-sets, a contingency table was calculated for its presence in the gene-set compared to the rest of the genome and the presence of the other COGs in the gene-set compared to rest of the genome. R 3.0.2 [72] was used to perform a Fisher’s exact test on each contingency table and to adjust *p*-values using the Benjamini-Hochberg method [73] of controlling the false discovery rate for multiple testing.

## Availability of supporting data

The multiple genome alignment and phylogenetic data are available from the Dryad Digital Repository: http://dx.doi.org/10.5061/. The sequencing data supporting the results of this article are available in the European Nucleotide Archive: [EMBL:ERS540821, EMBL:ERS540822, EMBL:ERS540823, EMBL:ERS540826, EMBL:ERS540825, EMBL:ERS540877, EMBL:ERS540807, EMBL:ERS540874, EMBL:ERS540827, EMBL:ERS540861, EMBL:ERS540860, EMBL:ERS540806, EMBL:ERS540824, EMBL:ERS540820, EMBL:ERS540875, EMBL:ERS540876, EMBL:ERS540837, EMBL:ERS540993, EMBL:ERS541232, EMBL:ERS605528, EMBL:ERP006877, EMBL:ERP006880, EMBL:ERP006881, EMBL:ERP006882, EMBL:ERP006883, EMBL:ERP006884, EMBL:ERP006885, EMBL:ERP006886, EMBL:ERP006887, EMBL:ERP006888, EMBL:ERP006889, EMBL:ERP006890, EMBL:ERP006891, EMBL:ERP006892, EMBL:ERP006893, EMBL:ERP006894, EMBL:ERP006895, EMBL:ERP006896, EMBL:ERP006897, EMBL:ERP006898, EMBL:ERP006899, EMBL:ERP006900, EMBL:ERP006901].

## Additional files

Additional file 1:
**Detected regions of recombination in all strains.** Information regarding the 21 detected regions of recombination across all examinied X. citri pv. citri strains, including information about the strains in which the event is detected, the genes contained within the recombination zone and functional information about the genes where available. (XLSX 23 kb) Additional file 2:
**Pathotype-specific regions of recombination and number of introduced variant residues in genes.** Information about the pathotype-specific recombination regions, including which genes are involved in each event, and the number of pathotype-specific variant residues found in each gene. “-” denotes cases where the number of pathotype-specific residues could not be calculated. (XLSX 10 kb) Additional file 3:
**Phylogeny from whole genome alignment of** ***X. citri*** **pv** ***. citri*** **strains containing regions of recombination.** A reconstructed phylogeny containing regions of recombination and with a distribution of A strains that contain recombination event 16. The phylogeny was reconstructed under the GTR model of nucleotide substitution. The pathotypes are colored as follows: A strains are dark blue, A^w^ strains are orange, A* strains are purple. (PDF 26 kb) Additional file 4:
**Table of best reciprocal blast hit relationships for genes across all examined strains.** Each strain in the dataset is represented as a column, and each row represents best reciprocal hits. The rows are ordered initially by the gene order from IAPAR 306, and then arbitrarily by different strains cycling through the A, Aw and A* pathotypes. The strains are highlighted based on their pathotype: A strains (blue), A^w^ strains (orange) and A* strains (purple). Additional information for certain genes are contained in parenthesis after the systematic gene name. (XLSX 2199 kb) Additional file 5:
**Genic and intergenic pathotype-specific SNPs.** Information about all identified pathotype-specific SNPs in the dataset, including the SNP location, the codon and amino acid change, and functional gene information where available. (XLSX 34 kb) Additional file 6:
**Pathotype-specific significantly enriched or depleted (** ***P*** **<0.05) COG categories for missing genes, SNP-containing genes or genes within zones of recombination.** (XLSX 12 kb) Additional file 7:
**Examination of putative pathotype-specific genes from Zhang et al. [** 17 **].** Each strain in the dataset is represented as a column, and each row represents best reciprocal hits to genes identified as either unique to A strains or unique to A^w^ and A* strains by Zhang et al. [17] with a description of their status from our dataset, their distribution across all strains in our dataset, and whether they coincide with detected regions of recombination. The strains are highlighted based on their pathotype: A strains (blue), A^w^ strains (orange) and A* strains (purple). (XLSX 63 kb) Additional file 8:
**Phylogeny reconstructed from non-tip SNPs in non-recombinant genomic regions for downstream ancestral character estimation.** The pathotypes are colored as follows: A strains are dark blue, A^w^ strains are orange, A* strains are purple and the shared A/A^w^ branch is yellow. (PDF 73 kb) Additional file 9:
**Analysis of pathotype-specific protein lengths.** The mean lengths, standard deviation and coefficient of variation of the orthologous proteins present in each pathotype are shown for cases where there is a difference in the mean length between the pathotypes and a small (< 0.05) coefficient of variation within a pathotype. (XLSX 11 kb)

## Abbreviations

BRH: best reciprocal hit
DSF: diffusable signal factor
GDE: glycerophosphoinositol phosphoDiEsterase
HR: hypersensitive response
MLVA: multi locus variable number of tandem repeat analysis
SNP: single nucleotide polymorphism
T3E: type III effector
