# Supplementary figures and images for: Comparative genomics of 43 strains of Xanthomonas citri pv. citri reveals the evolutionary events giving rise to pathotypes with different host ranges
Source: BMC Genomics. 2015 Dec 23;16:1098. doi: 10.1186/s12864-015-2310-x (PMC4690215; doi:10.1186/s12864-015-2310-x)

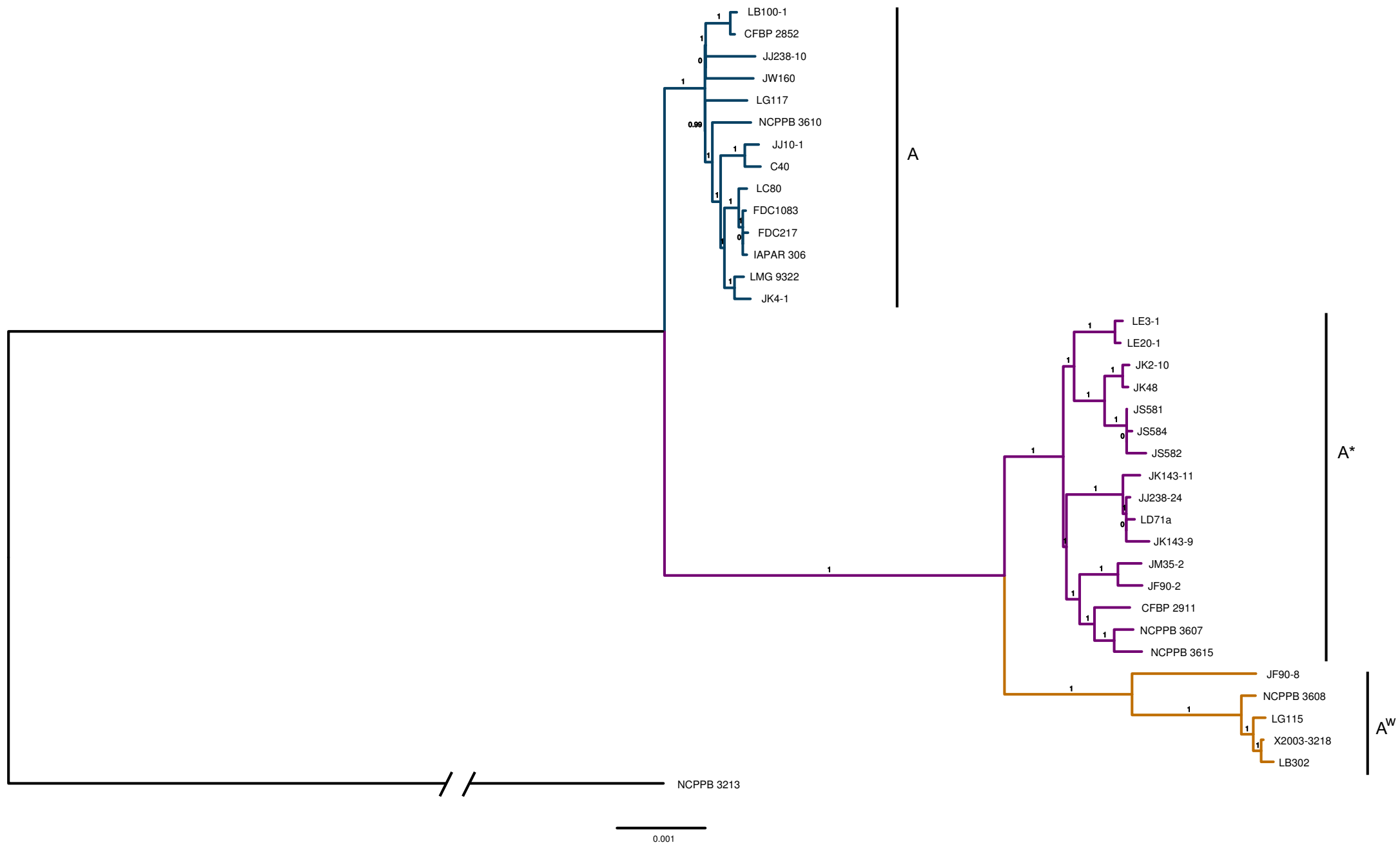

Supplement: Additional file 3: — Phylogeny from whole genome alignment of X. citri pv . citri strains containing regions of recombination. A reconstructed phylogeny containing regions of recombination and with a distribution of A strains that contain recombination event 16. The phylogeny was reconstructed under the GTR model of nucleotide substitution. The pathotypes are colored as follows: A strains are dark blue, Aw strains are orange, A* strains are purple. (PDF 26 kb) [file 12864_2015_2310_MOESM3_ESM.pdf]

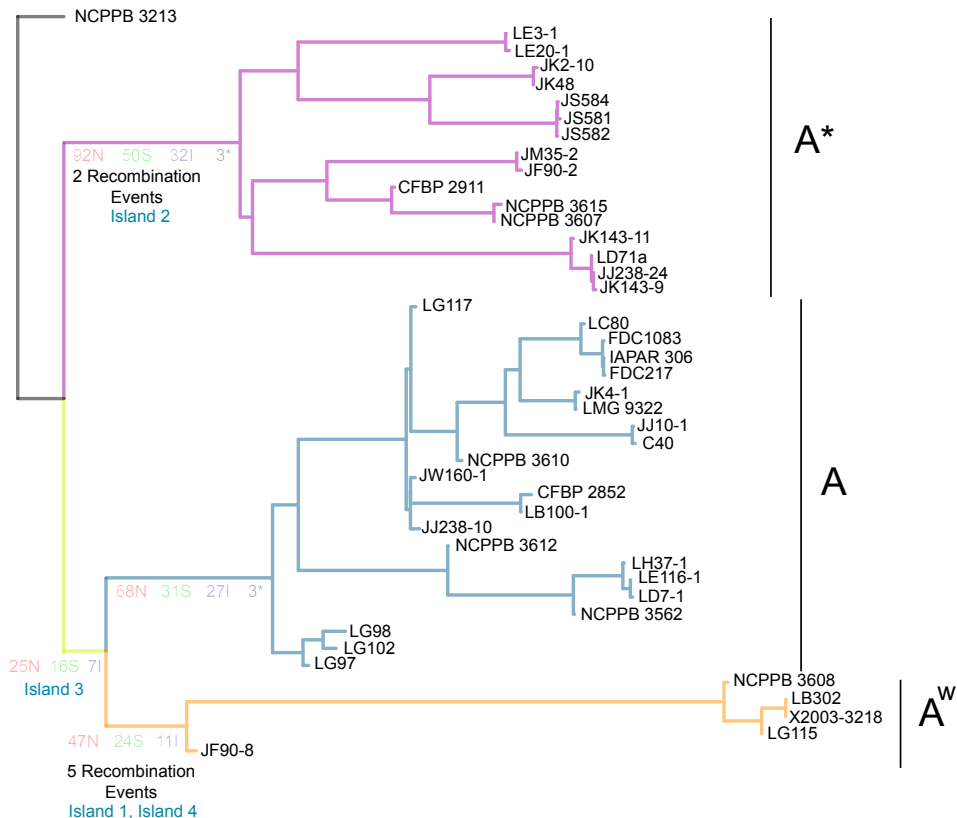

Supplement: Additional file 8: — Phylogeny reconstructed from non-tip SNPs in non-recombinant genomic regions for downstream ancestral character estimation. The pathotypes are colored as follows: A strains are dark blue, Aw strains are orange, A* strains are purple and the shared A/Aw branch is yellow. (PDF 73 kb) [file 12864_2015_2310_MOESM8_ESM.pdf]
